# Functional and Mood Outcomes in Bipolar Disorder Patients With and Without Substance Use Disorders Undergoing Psychotherapy

**DOI:** 10.3389/fpsyt.2021.661458

**Published:** 2021-09-14

**Authors:** William Moot, Marie Crowe, Maree Inder, Kate Eggleston, Christopher Frampton, Richard Porter

**Affiliations:** Department of Psychological Medicine, University of Otago, Christchurch, New Zealand

**Keywords:** bipolar disorder, function, mood, psychotherapy, substance usage disorders (SUDs)

## Abstract

**Objectives:** Research suggests that patients with co-morbid bipolar disorder (BD) and substance use disorder (SUD) have a poorer illness course and clinical outcome. The evidence is limited as SUD patients are often excluded from BD studies. In particular, evidence regarding long term outcomes from studies using psychotherapies as an adjunctive treatment is limited. We therefore examined data from two studies of Interpersonal Social Rhythm Therapy (IPSRT) for BD to determine whether lifetime or current SUD affected outcomes.

**Methods:** Data were analyzed from two previous clinical trials of IPSRT for BD patients. Change in scores on the Social Adjustment Scale (SAS) from 0 to 78 weeks and cumulative mood scores from 0 to 78 weeks, measured using the Life Interval Follow-Up Evaluation (LIFE), were analyzed.

**Results:** Of 122 patients (non-SUD *n* = 67, lifetime SUD but no current *n* = 43, current SUD *n* = 12), 79 received IPSRT and 43 received a comparison therapy—specialist supportive care—over 18 months. Lifetime SUD had a significant negative effect on change in SAS score but not LIFE score. There was no effect of current SUD on either change in score. Secondary analysis showed no correlation between symptom count and change in SAS total score or LIFE score.

**Conclusion:** Current SUD has no impact on mood or functional outcomes, however, current SUD numbers were small, limiting conclusions. Lifetime SUD appears to be associated with impaired functional outcomes from psychotherapy. There is limited research on co-morbid BD and SUD patients undergoing psychotherapy.

## Background

The prevalence of co-morbid Substance Use Disorder (SUD) in Bipolar Disorder (BD) has been shown to be consistently higher than in the general population. Both McElroy et al. (1) and Merikangas et al. (2) found that 42% of patients with BD had a lifetime history of SUD. Estimates from a large-scale trial and epidemiological studies found the rate of life-time co-morbid SUD in BD-I patients was between 52–61% (3–6) and 36.5–48% in BD-II (3, 6). The likelihood of having a lifetime co-morbid SUD in BD has been reported at 3–6 times greater than the general population (3, 7).

Patients with symptoms of BD are more likely to experience impaired occupational performance (8), disruption of relationships (9), and impaired cognitive functioning (10). This is exacerbated by substance use, and several cross-sectional studies (7, 11–13) have found greater baseline impairment in occupational, relationship and cognitive function in co-morbid BD and SUD patients, both current and with a lifetime history. It is also well-established that co-morbid SUD leads to poorer clinical outcomes including unstable course of illness (1, 14, 15) decreased quality of life (16) and increased suicide attempts (17). When comparing co-morbid SUD in Schizophrenia, Major Depressive Disorder (MDD) and BD patients, Marquez-Arrico and Adan found BD patients with co-morbid SUD were more likely to be emotionally upset, worried, fearful, lacking self-confidence, and more sensitive to criticism, than patients with Schizophrenia or MDD, and co-morbid SUD (18). Their study also confirmed previous findings of higher impulsivity and sensation seeking observed in patients with BD and co-morbid SUD. One study using the 36-Item Short Form Health Survey (SF-36) found BD patients with co-morbid SUD to have improved physical functioning when compared to co-morbid SUD in MDD and Schizophrenia, and vitality when compared to Schizophrenia. Findings also indicated significantly poorer health-related quality of life, especially Social Functioning, Role-Emotional and Mental Health, compared with population norms (19).

The traditional clinical emphasis on acute symptom reduction in BD has shifted to include longer-term focus on recovery of functioning in everyday life (20). Due to the high-level of functional and mood impairment in co-morbid BD and SUD, substance use-focused therapies have been suggested in this subpopulation (17, 21, 22). However, there is a paucity in structured trials of psychotherapy in this population and limited evidence regarding the most effective course of treatment.

Previous studies have examined possible risk factors for co-morbid BD and SUD which include earlier age of onset, male gender, presence of mixed mania, and family history of SUD (17, 23, 24). One potential mechanism for the higher rates of SUD in BD is that substance use provides a sense of “control” for patients, which provides stabilization of mood and daily rhythms (25, 26). Other suggested catalysts for substance use are sensation seeking (27), and heightened impulsivity (28). Healey et al. (29) proposed five categories of substance use in BD; experimenting in the early illness, living with serious mental illness, enjoying the effects of substances, feeling normal, and managing stress. However, the overrepresentation of SUD in BD is still not well-understood. Levin and Hennessy (30) surmised that while substance abuse may cause BD in predisposed patients, BD may also precipitate the onset of substance use in a number of patients.

A recent systematic review [Crowe et al. (31)] examined the efficacy of psychotherapy in co-morbid BD and SUD, identifying seven studies of psychotherapies in BD which included patients with co-morbid SUD. Two randomized controlled trials (RCTs) (32, 33) recruited only patients with co-morbid BD and current SUD and examined the effectiveness of Integrated Group Therapy (IGT), a treatment specifically developed to address this subpopulation. A first study (*n* = 62) found that compared with those receiving group drug counseling, patients receiving IGT had significantly fewer days of substance use. While mood symptoms decreased overall, patients receiving IGT had higher levels of depressive symptoms (32). A follow-up RCT (*n* = 61) re-designed the therapy for delivery in community settings (33). This study found a trend toward significance for reduction of substance use and no significant differences in mood symptoms (33). Secondary analyses showed a greater likelihood of abstinence and time to abstinence for IGT (33).

In patients who were all depressed at baseline and received one of several psychotherapies as part of the Systematic Treatment Enhancement Program for BD psychosocial treatment trial, Gold et al. (34) found that a current SUD was associated with an increased likelihood of recovery regardless of therapy type. Furthermore, they found that neither lifetime nor current SUD moderated the difference between “intensive” psychotherapy and much less intensive control treatments. The authors suggested that the better outcome in co-morbid SUD may relate to patients with SUD entering the study at a lower level of depressive severity or chronicity and therefore being more likely to recover quickly.

Two other psychotherapy studies have examined the impact of SUD on longer term outcome in BD. In a trial of IPSRT, Frank et al. (35) found that lifetime SUD had no effect on time to relapse but did not report on cumulative mood symptoms. Kallestad et al. (36) found that in patients with harmful substance use (HSU), those receiving group psychoeducation as opposed to individual psychoeducation had the shortest relapse time, shorter also than in patients without HSU. HSU was defined as a positive result on one of two screening tests [the Short Michigan Alcohol Screening Test (37) and the Drug Abuse Screening Test (38)] and may have been diagnosed at a lower level compared with Diagnostic and Statistical Manual of Mental Disorders (DSM) SUD.

In two previous RCTs examining psychotherapy for BD, we included patients with SUD, although those with severe alcohol and substance dependence were excluded, i.e., patients for who this was their primary problem requiring treatment at study entry (acute alcohol or substance dependence/abuse that required treatment, wherein symptoms of BD were clinically assessed to be a secondary issue). Results of these trials, of psychotherapy for bipolar disorder, have been published (39, 40). The RCTs examined the efficacy of Interpersonal and Social Rhythm Therapy (IPSRT) compared with Specialist Supportive Care (SSC) (39) and treatment as usual (TAU) (40). This has provided the opportunity to examine outcome in a larger number of patients receiving psychotherapy for BD. Here we report a secondary analysis of these studies, examining the effect of current and lifetime SUD on change in functioning and mood symptoms over 18 months of psychotherapy.

## Methods

Data are from patients in two randomized control trials (RCT) of IPSRT for BD referred to as Study 1 (39) and Study 2 (40). All patients who participated in 18 months of structured therapy during these trials were considered eligible for *post-hoc* combined analysis. Patients who received TAU were excluded from the analysis.

### Inclusion/Exclusion Criteria

In Study 1, patients were aged 15–36 years with BD-I, BD-II or BD not otherwise specified (defined as fulfilling the criteria for BD-II, with 2 days of hypomania). There were no criteria regarding mood state at entry. In Study 2, patients were patients with BD-I or BD-II aged 18–64 years, who did not meet the criteria for an episode of depression, mania, or mixed state at baseline. Exclusion criteria for both studies were minimal and included a primary diagnosis of schizophrenia, schizoaffective disorder, or the primary problem at the time was severe SUD. Severe SUD was defined as patients for who this was their primary problem requiring treatment at study entry (acute alcohol or substance dependence/abuse that required treatment, wherein symptoms of BD were clinically assessed to be a secondary issue). This exclusion was based on assessing clinician judgement and no quantitative cut-off was measured. Patients excluded on this basis were not recorded, but the primary study co-ordinators for both studies estimate the number of patients excluded for severe SUD diagnosis to be <5.

Differences between study designs that may have resulted in biased populations were the age difference (Study 1: 15–36 years, Study 2: 18–64 years) and mood state at baseline (Study 1: no criteria, Study 2: exclusion if assessed to be in episode).

### Assessment

The Structured Clinical Interview for DSM–IV Axis I Disorders (SCID-I) (41) and for Axis II Disorders (SCID-II) (42) were used to confirm psychiatric and substance use diagnoses. Patients had a current history of SUD if they were assessed on the SCID-I to have had an abuse or dependence on alcohol, amphetamines, cannabis, cocaine, hallucinogens, phencyclidine, opiates, and/or sedatives/hypnotics/anxiolytics. The SCID-I also allows patients to list other drugs and report symptoms of abuse or dependence. Lifetime history of SUD was defined as those who reported symptoms of abuse or dependence outside of the 30 days preceding initial assessment. Cumulative burden of mood symptoms was measured using the Longitudinal Interval Follow-up Examination (LIFE). The LIFE is used to retrospectively rate the severity of depression and mania on a weekly basis over the previous 6 months. Patients are rated on a 0–5 scale, where 0 relates to no symptoms and a score of 5 means that the patient fulfills definite criteria with the presence of either psychotic symptoms or extreme impairment in functioning (43). Ratings were carried out by a trained research assistant, by telephone, blind to treatment.

Patients completed the Social Adjustment Scale (SAS) which is a 45-item self-report questionnaire about social functioning over the previous 2 weeks. A score is derived from 11 subscale scores, which are averaged to give a final score in the range of 1–5, with a lower score reflecting greater social adjustment (44). Here, we examine change in SAS between baseline and 78 weeks.

### Psychotherapeutic Intervention

In both studies IPSRT was used and delivered according to a manualized protocol. IPSRT combines interpersonal psychotherapy with social rhythm therapy to help patients reduce stressors that may lead to relapse and to learn to live with bipolar disorder and its impact on their lives. The timing of sessions was flexible based on patient need, usually consisting of 10–12 weekly sessions, followed by 6–8 fortnightly sessions, and 4–5 monthly sessions thereafter, with a total of ~24 sessions.

In Study 1 patients were randomized to receive IPSRT or Specialist Supportive Care (SSC). SSC was designed as a control psychotherapy based on American Psychiatric Association (APA) guidelines (45) for the management of BD plus the core features of supportive psychotherapy. SSC combines supportive psychotherapy and psychoeducation, with the focus of each session initiated by the patient. It is not organized around a systematic exploration of interpersonal issues or social rhythms. All patients in this study were included in this analysis as they had received a form of psychotherapy for BD.

In Study 2 patients were randomized to IPSRT or Treatment as Usual (TAU). Patients randomly assigned to the TAU remained under care from their general practice physician and did not receive psychotherapy, and therefore were not analyzed in this study.

For all psychotherapy patients, treating psychiatrists made medication changes using clinical judgment and guided by a decision tree to optimize psychopharmacological treatment. Medication decisions were consistent with the APA (45) and Royal Australian and New Zealand College of Psychiatrists (RANZCP) Guidelines (20) for the treatment of BD.

### Ethics

Both studies gained ethical approval from the Canterbury Ethics Committee (Study 1) and New Zealand Health and Disability Commission (Study 2). They were registered prospectively with the Australia and New Zealand Clinical Trials Registry (Study 1—ACTRN12605000722695) (Study 2—ACTRN12611000961943).

### Primary Outcome Measures

In Study 1 the primary outcome was the cumulative burden of depressive symptoms as measured by the LIFE. Study 2 had two primary outcomes: time to relapse and readmission.

In this pooled analysis, outcome measures determined a priori were change in mood symptoms as measured by the LIFE and change in functional impairment as measured by the SAS.

### Statistical Analyses

Analyses used the Statistical Package for the Social Sciences (SPSS) version 25. Baseline demographic and clinical characteristics of the SUD and non-SUD groups were recorded. Independent *t*-tests for continuous variables and Chi-square tests for categorical variables were used to test for significant differences between the groups. Baseline demographic and clinical characteristics of the initial study treatment allocations were recorded. Between group differences were examined using Fisher's protected least significant difference test for continuous variables, and *post-hoc* Chi-square tests for categorical variables.

The primary analysis utilized a univariate general linear model. Dependent variables were LIFE score changes from 0–26 to 52–78 weeks (total/depression/mania) and change in SAS from baseline to 78 weeks. Study 2 did not measure the LIFE from baseline (i.e., for the 6 months before entry into the study) but conducted the first LIFE rating at 26 weeks (i.e., 0 to 26 weeks). Psychotherapy group (IPSRT study 1/SSC study 1/IPSRT study 2) and lifetime SUD were entered as fixed factors. Analyses were then adjusted independently and collectively for co-variates identified as significantly different or trending toward significant difference at baseline (age at onset of first BD episode, lifetime history of any anxiety disorder and relevant baseline score). All dependent variables were normally distributed.

A further analysis utilizing the same univariate general linear model was completed to examine the effect of current SUD, where patients were given a score of 0 (no lifetime history of SUD), 1 (lifetime but no current SUD) or 2 (current SUD).

A secondary analysis examined Spearman's correlations between SUD symptom count and the same dependent variables. SUD symptom count was identified as the highest score in any of substance or alcohol, dependence or withdrawal categories as measured by the DSM-IV SCID at week 0. Scores ranged nominally from 0 to 7, with a higher score denoting more experiences of SUD-related symptoms throughout their illness course prior to the study.

## Results

### Sample Characteristics

**Figure 1:**
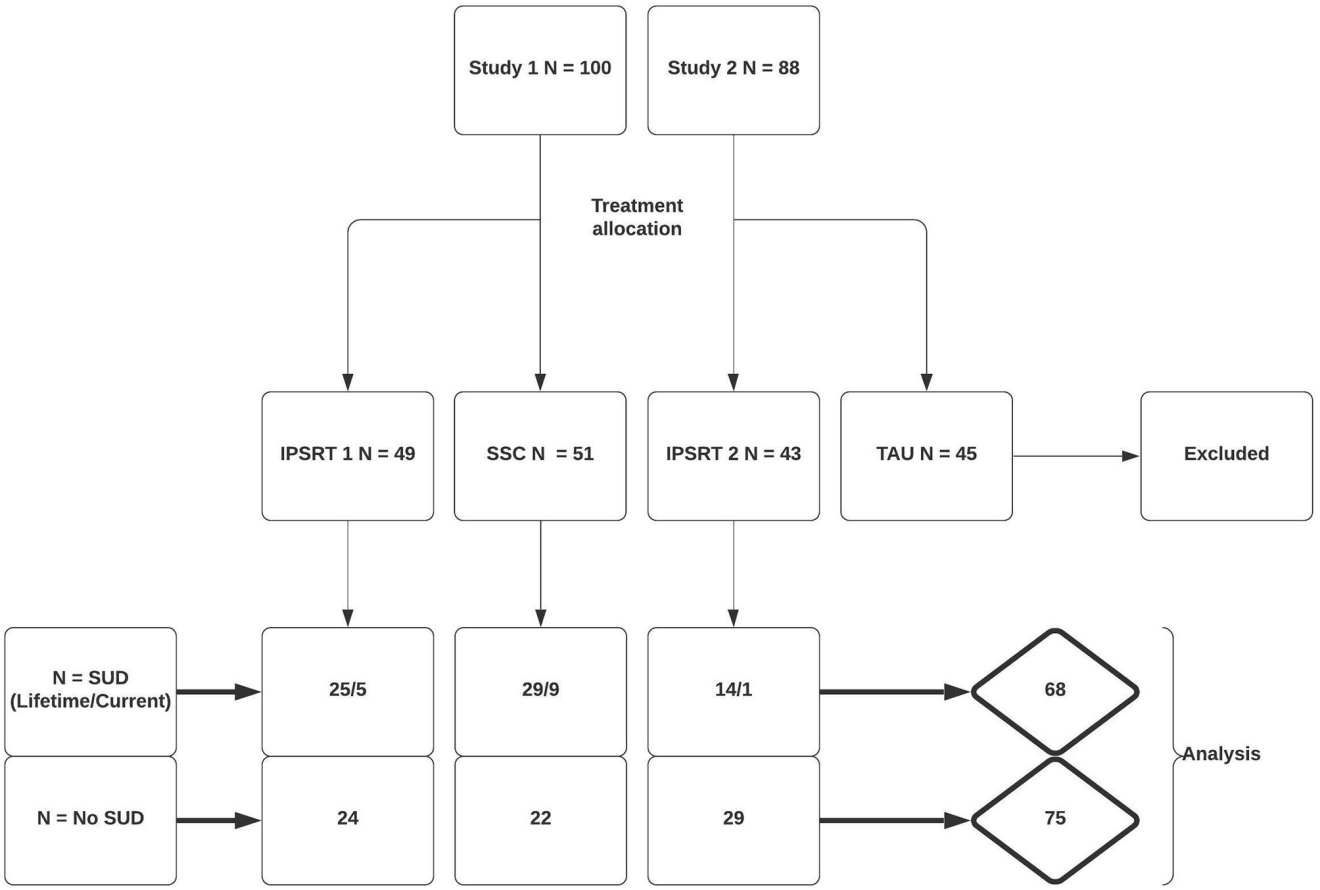
Flow diagram of patient allocation.

In Study 1 100 patients were randomized to IPSRT (*n* = 49) or SSC (*n* = 51). Eighty-one patients completed the study, 38 (78%) and 43 (86%) in each respective arm. In Study 2 88 patients were randomly assigned to IPSRT (*n* = 43) or to TAU (*n* = 45). In the IPSRT group, 41 patients were analyzed following drop-out (95%). The TAU group were not included in analysis. Therefore, 143 patients were included in the analyses. Patient allocation to initial treatment and break-down of patients with co-morbid SUD by treatment allocation is shown in Figure 1.

Baseline demographic and clinical characteristics of all patients grouped by lifetime SUD/non-SUD are presented in Table 1. Sixty-eight patients (47.6%) had a lifetime SUD. Mean age at onset was significantly lower in the lifetime SUD (mean 14.6 years; SD 4.5) compared with the non-SUD group (mean 16.8 years; SD 6.4) [*t*(141) = 2.35; *p* = 0.02]. The rate of a lifetime diagnosis of an anxiety disorder was significantly higher in patients with lifetime SUD (65%) than non-SUD (36%) (*p* = 0.001). Mean baseline functioning was not significantly different between groups; with mean SAS 2.3 (SD 0.6) in lifetime SUD and 2.2 (SD 0.5) in non-SUD [*t*_(139)_ = −1.90; *p* = 0.06]. Cumulative mood symptoms (LIFE scores between weeks 0 and 26) were higher in the lifetime SUD group (mean 1.9; SD 1.3) compared with the non-SUD group (mean LIFE week 0–26 = 1.5; SD 1.3) [*t*_(135)_ = −2.05; *p* = 0.04]. Depressive and manic mood scores did not differ between groups (*p* = 0.09). There was no difference in dropout rates in patients with lifetime SUD (*n* = 13) compared to those without (*n* = 8) (*p* = 0.23). The number of therapy sessions attended did not differ between groups (*p* = 0.63). Lifetime SUD patients were primarily made up of Cannabis (*n* = 58) and Alcohol (*n* = 53) consumers. Current SUD patients accounted for 14 of the lifetime SUD patients and followed a similar pattern with Cannabis (*n* = 13), followed by Alcohol (*n* = 6) and unspecified (*n* = 1) accounting for all patients in this subpopulation.

**Table 1:** Clinical characteristics of lifetime SUD and non-SUD groups.

	**No lifetime substance use disorder** ** (** ***N*** **=** **75)**		**Lifetime substance abuse disorder** ** (** ***N*** **=** **68)**			
**Characteristic**	***N* (%)**	**M ± SD**	***N* (%)**	**M ± SD**	**Effect size**	***p***
Age		31.7 ± 12.7		29.2 ± 9.1	0.23	0.17
Gender (F)	58 (77)		51 (75)		−0.27	0.74
Ethnicity (Pākehā)	63 (84)		53 (78)		0.18	0.10
Bipolar 1/2	54/17 (72/23)		52/15 (77/22)		0.11	0.44
Index episode (depressive)	64 (85)		60 (88)		0.16	0.15
Rapid cycling	16 (23)		23 (34)		0.13	0.09
Age at onset		16.8 ± 6.4		14.6 ± 4.5	0.39	0.02
Lifetime anxiety disorder	27 (36)		44 (65)		0.29	<0.001
Drugs of preference: Alcohol/Cannabis				53/58 (37/41)		
Medication use^†^						
Lithium	20 (27)		21 (31)		0.05	0.58
Anticonvulsant mood stabilizer	27 (36)		29 (43)		0.07	0.42
Antipsychotic	37 (49)		33 (49)		−0.01	0.92
Antidepressant	40 (53)		37 (54)		0.01	0.90
Therapy sessions completed		27.7 ± 11.0		26.8 ± 11.9	0.08	0.63
Drop-out	8 (11)		13 (19)		0.1	0.23
SAS total score^†^		2.2 ± 0.5		2.3 ± 0.6	−0.32	0.06
Cumulative mood score^‡^		1.5 ± 1.3		1.9 ± 1.3	−0.35	0.04
Depressive mood score^‡^		1.1 ± 1.0		1.4 ± 0.9	−0.30	0.09
Manic mood score^‡^		0.4 ± 0.5		0.5 ± 0.6	−0.29	0.09

† *At 0 weeks*.

‡ *Retrospective from 0 to 26 weeks*.

Baseline demographic and clinical characteristics of the treatment groups are presented in Table 2. Given Study 1 specifically recruited young patients (aged 15–35), as expected there was a difference in age between the study groups. In Study 2, patients were recruited out of episode resulting in a more well population at baseline. Baseline SAS and mood scores were significantly different from Study 2 (IPSRT 2) to Study 1 (IPSRT 1 and SSC). There were no other differences between the treatment groups.

**Table 2:** Clinical characteristics of study group.

	**IPSRT 1 (** ***N*** **=** **49)**		**SSC (** ***N*** **=** **51)**		**IPSRT 2 (** ***N*** **=** **43)**		
**Characteristic**	***N* (%)**	**M ± SD**	***N* (%)**	**M ± SD**	***N* (%)**	**M ± SD**	**Effect size**
Age		26.6 ± 5.9		26.5 ± 6.0		39.9 ± 14.4^**^	0.31
Gender (F)	37 (75)		39 (76)		33 (77)		
Ethnicity (Pākehā)	42 (86)		41 (80)		33 (77)		
Bipolar 1/2	37/7 (76/14)		41/10 (80/20)		28/15 (65/35)		
Index episode (depressive)	43 (88)		46 (90)		35 (81)		
Rapid cycling	16 (33)		15 (30)		8 (19)		
Age at onset		15.7 ± 4.8		14.6 ± 5.2		17.3 ± 6.7	
Lifetime anxiety disorder	28 (57)		30 (59)		13 (30)		
Lifetime/current SUD	25/5 (51/10)		29/9 (57/18)		14/1 (33/2)		
Medication use^†^							
Lithium	13 (27)		16 (31)		12 (28)		
Anticonvulsant mood stabilizer	18 (37)		20 (39)		18 (42)		
Antipsychotic	24 (49)		25 (49)		21 (49)		
Antidepressant	26 (53)		27 (53)		24 (56)		
Drop out	11 (22)		8 (16)		2^*^ (5)		0.21
SAS total score^†^		2.3 ± 0.6		2.3 ± 0.5		2.1 ± 0.5^*^	0.04
Cumulative mood score (LIFE)^‡^		2.1 ± 1.1		1.8 ± 1.4		1.1 ± 1.2^*^	0.11

† *At 0 weeks*.

‡ *Retrospective from 0 to 26 weeks*.

* *Significantly different from IPSRT 1 and SSC at p < 0.05*.

** *Significantly different from IPSRT 1 and SSC at p < 0.001*.

Twelve patients had a current SUD. No differences were found in clinical characteristics of this group compared with the rest of the sample.

### Outcomes

The primary analysis was of the impact of lifetime SUD on change in functioning (SAS at 0 and 78 weeks) and change in mood symptoms (LIFE at 26 and 78 weeks) over the course of the studies (see Table 3).

**Table 3:** Primary analyses—univariate ANOVA based on general linear model.

	**Unadjusted**		**Adjusted for relevant baseline score**		**Adjusted for baseline score, age at onset of first BD episode** **+** **history of anxiety disorder**	
	**SUD**	**SUD by treatment group**	**SUD**	**SUD by treatment group**	**SUD**	**SUD by treatment group**
Change in SAS total	*F*_(1, 116)_ = 1.65	*F*_(2, 116)_ = 3.25^*^	*F*_(1, 115)_ = 5.73^*^	*F*_(2, 115)_ = 1.30	*F*_(1, 113)_ = 5.58^*^	*F*_(2, 113)_ = 1.39
Change in Cumulative Affective Mean Score	*F*_(1, 121)_ = 0.01	*F*_(2, 121)_ = 0.35	*F*_(1, 120)_ = 1.21	*F*_(2, 120)_ = 0.15	*F*_(1, 118)_ = 0.42	*F*_(2, 118)_ = 0.32
Change in Depressive Mean Score	*F*_(1, 121)_ = 0.01	*F*_(2, 121)_ = 0.25	*F*_(1, 120)_ = 0.50	*F*_(2, 120)_ = 0.04	*F*_(1, 118)_ = 0.02	*F*_(2, 118)_ = 0.01
Change in Manic Mean Score	*F*_(1, 121)_ = 0.11	*F*_(2, 121)_ = 0.84	*F*_(1, 120)_ = 2.38	*F*_(2, 120)_ = 1.37	*F*_(1.118)_ = 1.90	*F*_(2, 118)_ = 1.40

* *Significant at P < 0.05*.

Initially there was no significant difference between lifetime SUD and non-SUD groups for change in SAS Total. However, after adjusting for SAS total at week 0, a significant difference was found (*F* = 5.73; *p* = 0.02) which was still present after adjusting for further variables (*F* = 5.58; *p* = 0.02) (see Table 3). Table 4 demonstrates the difference in estimated mean change in SAS Total.

**Table 4:** Primary analyses—estimated marginal means and confidence intervals for change in SAS total.

	**No SUD**		**SUD**		
**Outcome measure**	**EMM**	**CI**	**EMM**	**CI**	***P*-value**
Change in SAS total	0.36	0.22–0.49	0.22	0.07–0.38	0.20
Change in SAS total adjusted for baseline SAS	0.39	0.28–0.50	0.19	0.07–0.31	0.02
Change in SAS total adjusted for baseline SAS, age of onset of first BD episode, and history of any anxiety disorder	0.39	0.28–0.50	0.19	0.06–0.31	0.02

There was also a significant interaction between lifetime SUD and treatment group for change in SAS scores (*F* = 3.25; *p* = 0.04). However, after adjusting for variables that were significantly different between groups at baseline (SAS score, age of onset of first BD episode and lifetime history of any anxiety disorder), the treatment by SUD interaction became non-significant.

For change in mood symptoms as scored by LIFE, no significant effects of treatment group, lifetime SUD, or interaction between them were found. This was maintained for cumulative and independent (depressive/manic) scores across unadjusted and adjusted analysis.

When SUD was entered as a three-group factor (current SUD/lifetime SUD/no SUD) there was no effect of current SUD, or interaction with treatment group.

The median number of SUD symptoms suffered from in the lifetime SUD group was 4 (Interquartile range = 3–6). There were no significant correlations (p > 0.05) between lifetime SUD symptom count and changes in functioning (r = 0.23) or mood symptoms (Cumulative r = 0.14, Depressive r = −0.11, Manic r = 0.10).

## Discussion

This study examining the effect of current and lifetime SUD on change in functioning and mood symptoms over 18 months of psychotherapy found that patients with lifetime SUD had less improvement in functioning over 18 months of therapy than did those without lifetime SUD. However, there was no effect of lifetime SUD on change in cumulative mood symptoms. Additionally, there was no interaction between lifetime SUD and psychotherapy group for any variable, indicating that these findings were not dependent on the type of psychotherapy received. A current diagnosis of SUD did not impact on functioning or mood outcomes, but conclusions regarding this are less certain given the very low numbers of patients who met criteria for a current SUD. Similarly, there were no significant correlations between lifetime SUD symptom count and differential outcomes. However, this analysis is limited by the fact that symptom count is based on patient's recollection of symptoms when they suffered from SUD, which for most was not current.

Less positive functional outcomes related to 18 months of stabilization with intensive psychotherapy and concomitant pharmacotherapy in this population is of interest. We hypothesize that patients with a lifetime SUD but no current SUD may have a predisposition to experience a more severe illness course, and therefore be more resistant to functional improvements. It is also possible that residual cognitive impairment from previous substance use may play a role. However, there is limited extant literature to suggest why patients with a lifetime SUD might be resistant to functional improvements. We did not assess the recency of each patient's substance use for those with a lifetime SUD. Some patients may have had very recent SUD resulting in significant functional impairment which may not have been addressed during psychotherapy. Indeed, the baseline SAS score was higher in the SUD group, although only at a trend level.

The rate of co-morbid lifetime SUD in BD in our study was 47.5%. While data for New Zealand rates of lifetime SUD in BD is not available, this falls in line with rates reported by international epidemiological studies (3–6). It must be noted that these rates differ by country, for example, one BD study in South Korea found that just 17% of all BD patients had a lifetime history of SUD (46). The implications of regional differences of co-morbid lifetime SUD in BD are not clear.

In our sample, we found that patients with lifetime SUD had a higher rate of lifetime history of any anxiety disorder. Patients with a history of SUD are more likely to experience a co-morbid anxiety disorder than those without, for example in New Zealand, 40% of patients with a 12-month history of SUD have a co-morbid anxiety disorder, compared with a population anxiety rate of 14.8% (47). The reason for this is unclear but may relate to self-medication for anxiety symptoms or increased substance use being causative for anxiety (48). In this study there was a high rate (65%) of co-morbid lifetime anxiety disorders in the lifetime SUD group, compared with previously reported international rates of 30–40% (49, 50). The high rate of co-morbid anxiety disorders in our lifetime SUD sample is of interest. We hypothesize that the combination of lifetime SUD and anxiety disorder may make patients more likely to enter clinical trials of psychotherapy for BD, either because they or their clinicians believe this would be a potentially beneficial treatment. Interestingly, epidemiological studies done in both New Zealand and Australia showed 49.6 and 58.5% of patients, respectively, with any affective disorder had a co-morbid anxiety disorder (47, 51). However, there is limited evidence to support any speculation on increased rates of co-morbid anxiety disorders in our study population. The increased rate of anxiety disorder may also contribute to a poorer functional outcome and was therefore included as a co-variate in our model.

Individuals with co-morbid BD and SUD are more likely to experience more severe and longer affective episodes (11, 52) which are associated with higher levels of functional impairment (9, 53). However, there is very little previous data on the effects of psychotherapy on functioning and mood in patients with co-morbid BD and SUD, despite consistent evidence that patients with lifetime and current SUD have significantly poorer functioning and illness course (7, 11–13). A recent systematic review (31) of studies of psychotherapy for co-morbid BD and SUD patients found mixed results for mood outcomes and also found that no trials examined differential functional outcomes.

Crowe et al. (31) in their review of the psychotherapy for BD with co-morbid SUD, examined seven clinical trials; 2 IPSRT (35, 39), 2 IGT (32, 33), 1 STEP-BD intensive psychotherapy [Cognitive Behavioral Therapy (CBT), IPSRT, Family Focused Therapy or collaborative care] (34), CBT (54), and a psychoeducation intervention (36). The heterogeneity of treatment likely contributed to the contrasting results across these studies. Furthermore, there were differences in inclusion criteria related to mood state between studies. Frank et al. (35) required that participants had had three or more previous mood episodes and be in episode at baseline. The sub-analysis by Gold et al. (34) was based on patients meeting criteria for a current major depressive episode at entry. Inder et al. (39) recruited patients in any mood state. Kallestad et al. (36) required on-going treatment for BD as an entry requirement. Scott et al. (54) required patients to have had 2 or more episodes, with one having occurred over the past 12 months. Both IGT studies (32, 33) required patients to have a co-morbid SUD diagnosis at entry. Patient numbers at entry ranged from 85 (36) to 270 (34) and study periods ranged from 12 weeks (33) up to 2 years (36). Outcome measures were not consistent across studies and therefore made comparison and validation of study conclusions difficult. The review concluded that while the evidence is very limited, there was little evidence that people with comorbid diagnoses of BD and current low-moderate SUD symptoms may respond at least equally compared to those without SUD when treated with intensive individual or group psychotherapies.

The presence of co-morbid SUD is an important issue since BD patients are 3–6 times more likely to suffer from SUD during their lifetime than the general population. It is important to ascertain whether this affects outcome, and psychotherapies may need to be modified for delivery to patients with co-morbid SUD. Most previous psychotherapy for BD studies have excluded patients with co-morbid SUD, therefore there is very little evidence regarding the differential impact of psychotherapy in patients with co-morbid SUD. By pooling data from two studies, we were able to analyze outcomes from a larger group of co-morbid BD and lifetime SUD patients undergoing psychotherapy and have the potential to show clinically important differences. We also had a low dropout rate with only 15% over an 18-month intervention and examined longer term measures of cumulative mood, as well as measuring functioning which is crucial for patients' ongoing quality of life.

## Limitations

This secondary analysis of two RCTs for BD has several limitations. Firstly, it should be noted that the studies were not designed to be pooled. Each had different inclusion and exclusion criteria and primary outcome measures. In Study 1, patients were younger (Study 1: 15–36 years, Study 2: 18–64 years) and more likely to be unwell at baseline (Study 1: no criteria regarding mood state at study entry, Study 2: exclusion if assessed to be in episode). However, these differences were adjusted for in our analyses and meant that the sample had a broader range of characteristics and may be able to be generalized to a wider range of patients. Secondly, both studies excluded patients with a diagnosis of severe SUD, and although milder substance abuse or dependence were not excluded, relatively few patients (10% of the total sample) with current SUD were recruited. One possible explanation is that referring clinicians and patients perceived that long-term psychotherapy was less appropriate in the context of a current SUD. An epidemiological study of mental health in New Zealand showed that 12.9% patients with a 12-month history of any mood disorder had a co-occurring SUD (47). The percentage of patients with current SUD is also low in previous research, with Gold et al. (34) for example reporting current SUD in only 17.4% (*n* = 47) of their BD patients. Therefore, it is likely that our current sample reflects rates of co-morbid SUD in BD in New Zealand. However, it is important to note that lifetime SUD outcomes are not reflective of current SUD outcomes—patients with a current SUD are likely to have greater baseline functional and mood impairment (7, 13) which may influence results. Thirdly, SUD outcomes were not measured. Furthermore, we did not evaluate age of SUD onset, years of SUD or quantitative measures of substance consumption, giving us limited insight into the severity of SUD experienced both current and lifetime. This has been a limitation of most of the similar previous studies and it is clearly important in future research to measure these as well as mood related and functional outcomes. Fourthly, it has been noted that drug consumption is associated with a lack of regularity in circadian rhythms (55), as is Bipolar Disorder (56). Both studies analyzed did not assess patients' hourly habits (sleep-watch, activity), despite circadian rhythm regulation being a key component of IPSRT. It has been suggested that therapeutic approaches to addiction should take into account circadian rhythmic organization. In many cases, establishing regular time patterns of wake–sleep, meals and daily activity with a tendency toward a morningness pattern of functioning may suffice to regulate circadian rhythms in this population (57). Finally, the retrospective analysis meant that the trials had different measures. In particular, study 2 had LIFE data only from the 26-week point. Therefore, we were only able to analyze data from this point rather than from a baseline which would have included weekly ratings from 6 months prior to entry into the study. However, we note that the 26-week LIFE does extend back to week zero.

## Conclusion

In summary, in this *post-hoc* combined analysis of 122 BD patients undergoing 18 months of psychotherapy and medication management, there was a difference in change in functioning but not in mood outcomes over 78 weeks in patients with a lifetime history of SUD compared to those without SUD. This finding is broadly in line with the previous literature. Additionally, there was no difference in outcome for patients with current SUD, but the numbers of these patients were small. There are very few studies that have included significant numbers of patients with co-morbid current SUD, limiting understanding of the best treatment for this group. Numbers may be limited by expectations of patients and referrers regarding whether psychotherapies are appropriate in the context of co-morbid SUD. We recommend that studies include patients with current SUD and examine ways of increasing their recruitment and that SUD outcomes should be examined as well as mood and functioning.

## Data Availability Statement

The raw data supporting the conclusions of this article will be made available by the authors, without undue reservation.

## Ethics Statement

The studies involving human participants were reviewed and approved by New Zealand Health and Disability Commission Canterbury Ethics Committee. The patients/participants provided their written informed consent to participate in this study.

## Author Contributions

WM analyzed the data and wrote the first draft. RP supervised analysis and writing. CF supervised analysis. MC, MI, and KE were involved in planning of the analysis. All authors contributed to subsequent drafts.

## Funding

Both Study 1 and Study 2 were funded by grants from the Health Research Council of New Zealand.

## Conflict of Interest

RP had use of computer software at no cost for research—provided by SBT-pro. Received support for travel to educational meetings from Servier and Lundbeck. The remaining authors declare that the research was conducted in the absence of any commercial or financial relationships that could be construed as a potential conflict of interest.

## Publisher's Note

All claims expressed in this article are solely those of the authors and do not necessarily represent those of their affiliated organizations, or those of the publisher, the editors and the reviewers. Any product that may be evaluated in this article, or claim that may be made by its manufacturer, is not guaranteed or endorsed by the publisher.

## References

[1] McElroy SL Altshuler LL Suppes T Keck PE Frye MA Denicoff KD. Axis I psychiatric comorbidity and its relationship to historical illness variables in 288 patients with bipolar disorder. Am J Psychiat. (2001) 158:420–6. 10.1176/appi.ajp.158.3.420 11229983

[2] Merikangas KR Akiskal HS Angst J Greenberg PE Hirschfeld RMA Petukhova M. Lifetime and 12-month prevalence of bipolar spectrum disorder in the national comorbidity survey replication. Arch Gen Psychiat. (2007) 64:543–52. 10.1001/archpsyc.64.5.543 17485606 PMC1931566

[3] Regier DA Farmer ME Rae DS Locke BZ Keith SJ Judd LL. Comorbidity of mental-disorders with alcohol and other drug-abuse - results from the epidemiologic catchment-area (Eca) study. J Am Med Assoc. (1990) 264:2511–8. 10.1001/jama.264.19.2511 2232018

[4] Cassidy F Ahearn EP Carroll BJ. Substance abuse in bipolar disorder. Bipolar Disord. (2001) 3:181–8. 10.1034/j.1399-5618.2001.30403.x 11552957

[5] Grant BF Stinson FS Hasin DS Dawson DA Chou SP Ruan WJ. Prevalence, correlates, and comorbidity of bipolar I disorder and axis I and II disorders: results from the National Epidemiologic Survey on Alcohol and Related Conditions. J Clin Psychiatry. (2005) 66:1205–15. 10.4088/JCP.v66n1001 16259532

[6] Merikangas KR Jin R He JP Kessler RC Lee S Sampson NA. Prevalence and correlates of bipolar spectrum disorder in the world mental health survey initiative. Arch Gen Psychiat. (2011) 68:241–51. 10.1001/archgenpsychiatry.2011.12 21383262 PMC3486639

[7] Lagerberg TV Andreassen OA Ringen PA Berg AO Larsson S Agartz I. Excessive substance use in bipolar disorder is associated with impaired functioning rather than clinical characteristics, a descriptive study. BMC Psychiatry. (2010) 10:1–9. 10.1186/1471-244X-10-9 20105311 PMC2824653

[8] Simon GE Ludman EJ Unutzer J Operskalski BH Bauer MS. Severity of mood symptoms and work productivity in people treated for bipolar disorder. Bipolar Disord. (2008) 10:718–25. 10.1111/j.1399-5618.2008.00581.x 18837866

[9] Altshuler LL Post RM Black DO Keck PE Nolen WA Frye MA. Subsyndromal depressive symptoms are associated with functional impairment in patients with bipolar disorder: results of a large, multisite study. J Clin Psychiat. (2006) 67:1551–60. 10.4088/JCP.v67n1009 17107246

[10] Roux P Raust A Cannavo AS Aubin V Aouizerate B Azorin JM. Cognitive profiles in euthymic patients with bipolar disorders: results from the FACE-BD cohort. Bipolar Disord. (2017) 19:146–53. 10.1111/bdi.12485 28421717

[11] Cardoso TA Jansen K Zeni CP Quevedo J Zunta-Soares G Soares JC. Clinical outcomes in children and adolescents with bipolar disorder and substance use disorder comorbidity. J Clin Psychiatry. (2017) 78:e230–3. 10.4088/JCP.15m10293 28068464

[12] Cardoso Tde A Mondin TC Souza LD da Silva RA Magalhaes PV Kapczinski F. Functioning in bipolar disorder with substance abuse/dependence in a community sample of young adults. J Affect Disord. (2015) 187:179–82. 10.1016/j.jad.2015.08.046 26339928

[13] Jaworski F Dubertret C Ades J Gorwood P. Presence of co-morbid substance use disorder in bipolar patients worsens their social functioning to the level observed in patients with schizophrenia. Psychiatry Res. (2011) 185:129–34. 10.1016/j.psychres.2010.06.005 20587365

[14] Mitchell JD Brown ES Rush AJ. Comorbid disorders in patients with bipolar disorder and concomitant substance dependence. J Affect Disord. (2007) 102:281–7. 10.1016/j.jad.2007.01.005 17291591 PMC2735053

[15] Judd LL Akiskal HS Schettler PJ Endicott J Maser J Solomon DA. The long-term natural history of the weekly symptomatic status of bipolar I disorder. Arch Gen Psychiat. (2002) 59:530–7. 10.1001/archpsyc.59.6.530 12044195

[16] Singh J Mattoo SK Sharan P Basu D. Quality of life and its correlates in patients with dual diagnosis of bipolar affective disorder and substance dependence. Bipolar Disord. (2005) 7:187–91. 10.1111/j.1399-5618.2004.00173.x 15762860

[17] Messer T Lammers G Muller-Siecheneder F Schmidt RF Latifi S. Substance abuse in patients with bipolar disorder: a systematic review and meta-analysis. Psychiatry Res. (2017) 253:338–50. 10.1016/j.psychres.2017.02.067 28419959

[18] Marquez-Arrico JE Adan A. Personality in patients with substance use disorders according to the co-occurring severe mental illness: a study using the alternative five factor model. Pers Indiv Differ. (2016) 97:76–81. 10.1016/j.paid.2016.03.028

[19] Adan A Marquez-Arrico JE Gilchrist G. Comparison of health-related quality of life among men with different co-existing severe mental disorders in treatment for substance use. Health Qual Life Out. (2017) 15:209. 10.1186/s12955-017-0781-y 29061151 PMC5654090

[20] Malhi GS Bassett D Boyce P Bryant R Fitzgerald PB Fritz K. Royal Australian and New Zealand College of Psychiatrists clinical practice guidelines for mood disorders. Aust Nz J Psychiat. (2015) 49:1087–206. 10.1177/0004867415617657 26643054

[21] Salloum IM Thase ME. Impact of substance abuse on the course and treatment of bipolar disorder. Bipolar Disord. (2000) 2:269–80. 10.1034/j.1399-5618.2000.20308.x 11249805

[22] Juel A Kristiansen CB Madsen NJ Munk-Jorgensen P Hjorth P. Interventions to improve lifestyle and quality-of-life in patients with concurrent mental illness and substance use. Nord J Psychiat. (2017) 71:197–204. 10.1080/08039488.2016.1251610 27834103

[23] Tohen M Greenfield SF Weiss RD Zarate CA Vagge LM. The effect of comorbid substance use disorders on the course of bipolar disorder: a review. Harvard Rev Psychiat. (1998) 6:133–41. 10.3109/10673229809000321 10372281

[24] Goldstein BI Strober M Axelson D Goldstein TR Gill MK Hower H. Predictors of first-onset substance use disorders during the prospective course of bipolar spectrum disorders in adolescents. J Am Acad Child Adolesc Psychiatry. (2013) 52:1026–37. 10.1016/j.jaac.2013.07.009 24074469 PMC3787940

[25] Stalman MN Canham SL Mahmood A King D O'Rourke N. Aspects of control and substance use among middle-aged and older adults with bipolar disorder. Int J Ment Health Nurs. (2018) 27:833–40. 10.1111/inm.12371 28752582

[26] Gupta AK B NS Ghosh A Basu D. The link between bipolarity and substance use: a controlled clinic based study. Asian J Psychiatr. (2020) 47:101835. 10.1016/j.ajp.2019.10.015 31731145

[27] Bizzarri JV Sbrana A Rucci P Ravani L Massei GJ Gonnelli C. The spectrum of substance abuse in bipolar disorder: reasons for use, sensation seeking and substance sensitivity. Bipolar Disord. (2007) 9:213–20. 10.1111/j.1399-5618.2007.00383.x 17430295

[28] Nery FG Hatch JP Glahn DC Nicoletti MA Monkul ES Najt P. Temperament and character traits in patients with bipolar disorder and associations with comorbid alcoholism or anxiety disorders. J Psychiatr Res. (2008) 42:569–77. 10.1016/j.jpsychires.2007.06.004 17675066 PMC2693238

[29] Healey C Peters S Kinderman P McCracken C Morriss R. Reasons for substance use in dual diagnosis bipolar disorder and substance use disorders: a qualitative study. J Affect Disord. (2009) 113:118–26. 10.1016/j.jad.2008.05.010 18571735

[30] Levin FR Hennessy G. Bipolar disorder and substance abuse. Biol Psychiatry. (2004) 56:738–48. 10.1016/j.biopsych.2004.05.008 15556118

[31] Crowe M Eggleston K Douglas K Porter RJ. Effects of psychotherapy on comorbid bipolar disorder and substance use disorder: a systematic review. Bipolar Disord. (2020) 23:141–51. 10.1111/bdi.12971 32615028

[32] Weiss RD Griffin ML Kolodziej ME Greenfield SF Najavits LM Daley DC. A randomized trial of integrated group therapy versus group drug counseling for patients with bipolar disorder and substance dependence. Am J Psychiat. (2007) 164:100–7. 10.1176/ajp.2007.164.1.100 17202550

[33] Weiss RD Griffin ML Jaffee WB Bender RE Graff FS Gallop RJ. A “community-friendly” version of integrated group therapy for patients with bipolar disorder and substance dependence: a randomized controlled trial. Drug Alcohol Depend. (2009) 104:212–9. 10.1016/j.drugalcdep.2009.04.018 19573999 PMC2735139

[34] Gold AK Peters AT Otto MW Sylvia LG Magalhaes P Berk M. The impact of substance use disorders on recovery from bipolar depression: results from the Systematic Treatment Enhancement Program for Bipolar Disorder psychosocial treatment trial. Aust N Z J Psychiatry. (2018) 52:847–55. 10.1177/0004867418788172 30047784 PMC6778400

[35] Frank E Kupfer DJ Thase ME Mallinger AG Swartz HA Fagiolini AM. Two-year outcomes for interpersonal and social rhythm therapy in individuals with bipolar I disorder. Arch Gen Psychiat. (2005) 62:996–1004. 10.1001/archpsyc.62.9.996 16143731

[36] Kallestad H Wullum E Scott J Stiles TC Morken G. The long-term outcomes of an effectiveness trial of group versus individual psychoeducation for bipolar disorders. J Affect Disord. (2016) 202:32–8. 10.1016/j.jad.2016.05.043 27253214

[37] Selzer ML Vinokur A Vanrooijen L. Self-administered short michigan alcoholism screening-test (Smast). J Stud Alcohol. (1975) 36:117–26. 10.15288/jsa.1975.36.117 238068

[38] Skinner HA. The drug-abuse screening-test. Addict Behav. (1982) 7:363–71. 10.1016/0306-4603(82)90005-3 7183189

[39] Inder ML Crowe MT Luty SE Carter JD Moor S Frampton CM. Randomized, controlled trial of Interpersonal and Social Rhythm Therapy for young people with bipolar disorder. Bipolar Disord. (2015) 17:128–38. 10.1111/bdi.12273 25346391

[40] Crowe M Porter R Inder M Carlyle D Luty S Lacey C. Clinical effectiveness trial of adjunctive interpersonal and social rhythm therapy for patients with bipolar disorder. Am J Psychother. (2020) 73:107–14. 10.1176/appi.psychotherapy.20190035 32306747

[41] Spitzer RL Williams JBW Gibbon M First MB. The structured clinical interview for Dsm-Iii-R (Scid).1. History, rationale, and description. Arch Gen Psychiat. (1992) 49:624–9. 10.1001/archpsyc.1992.01820080032005 1637252

[42] Williams JBW Gibbon M First MB Spitzer RL Davies M Borus J. The structured clinical interview for Dsm-Iii-R (Scid).2. Multisite test-retest reliability. Arch Gen Psychiat. (1992) 49:630–6. 10.1001/archpsyc.1992.01820080038006 1637253

[43] Keller MB Lavori PW Friedman B Nielsen E Endicott J Mcdonaldscott P. The longitudinal interval follow-up evaluation - a comprehensive method for assessing outcome in prospective longitudinal-studies. Arch Gen Psychiat. (1987) 44:540–8. 10.1001/archpsyc.1987.01800180050009 3579500

[44] Weissman MM Bothwell S. Assessment of social adjustment by patient self-report. Arch Gen Psychiat. (1976) 33:1111–5. 10.1001/archpsyc.1976.01770090101010 962494

[45] American Psychiatric Association. Practice guideline for the treatment of patients with bipolar disorder. Am J Psychiat. (1994) 151:1–36. 10.1176/ajp.151.12.1 7977902

[46] Baek JH Cha B Moond E Ha TH Chang JS Kim JH. The effects of ethnic, social and cultural factors on axis I comorbidity of bipolar disorder: results from the clinical setting in Korea. J Affect Disorders. (2014) 166:264–9. 10.1016/j.jad.2014.05.027 25012440

[47] Scott KM McGee MA Browne MAO Wells JE Res NZMHS. Mental disorder comorbidity in Te Rau Hinengaro: the New Zealand Mental Health Survey. Aust N Z J Psychiat. (2006) 40:875–81. 10.1080/j.1440-1614.2006.01906.x 16959013

[48] Goodwin RD Stayner DA Chinman MJ Wu P Tebes JK Davidson L. The relationship between anxiety and substance use disorders among individuals with severe affective disorders. Compr Psychiat. (2002) 43:245–52. 10.1053/comp.2002.33500 12107861

[49] Baethge C Baldessarini RJ Khalsa HMK Hennen J Salvatore P Tohen M. Substance abuse in first-episode bipolar I disorder: indications for early intervention. Am J Psychiat. (2005) 162:1008–11. 10.1176/appi.ajp.162.5.1008 15863809

[50] Bauer MS Altshuler L Evans DR Beresford T Williford WO Hauger R. Prevalence and distinct correlates of anxiety, substance, and combined comorbidity in a multi-site public sector sample with bipolar disorder. J Affect Disord. (2005) 85:301–15. 10.1016/j.jad.2004.11.009 15780700

[51] Sweeney K Shui H Calder R Duggan M. The Economic Cost of Serious Mental Illness and Comorbidities in Australia and New Zealand. Melbourne, VIC: Royal Australian and New Zealand College of Psychiatrists; Australian Health Policy Collaboration (2016).

[52] Mammen G Rueda S Roerecke M Bonato S Lev-Ran S Rehm J. Association of cannabis with long-term clinical symptoms in anxiety and mood disorders: a systematic review of prospective studies. J Clin Psychiatry. (2018) 79:17. 10.4088/JCP.17r11839 29877641

[53] Simon GE Bauer MS Ludman EJ Operskalski BH Unurtzer J. Mood symptoms, functional impairment, and disability in people with bipolar disorder: specific effects of mania and depression. J Clin Psychiat. (2007) 68:1237–45. 10.4088/JCP.v68n0811 17854249

[54] Scott J Paykel E Morriss R Bentall R Kinderman P Johnson T. Cognitive-behavioural therapy for severe and recurrent bipolar disorders - randomised controlled trial. Brit J Psychiat. (2006) 188:313–20. 10.1192/bjp.188.4.313 16582056

[55] Kosobud AEK Gillman AG Leffel JK Pecoraro NC Rebec GV Timberlake W. Drugs of abuse can entrain circadian rhythms. Sci World J. (2007) 7:838545. 10.1100/tsw.2007.234 17982594 PMC5901354

[56] Alloy LB Ng TH Titone MK Boland EM. Circadian rhythm dysregulation in bipolar spectrum disorders. Curr Psychiat Rep. (2017) 19:21. 10.1007/s11920-017-0772-z 28321642 PMC6661150

[57] Adan A. A chronobiological approach to addiction. J Subst Use. (2013) 18:171–83. 10.3109/14659891.2011.63206028894915

